# Mapping of pain curricula across health professions programs at the University of Toronto

**DOI:** 10.1080/24740527.2018.1479841

**Published:** 2018-07-19

**Authors:** Laura Murphy, Leila Lax, Renata Musa, Sylvia Langlois, Sharona Kanofsky, Judith Hunter, Dinesh Kumbhare, Sara Promislow, Jon Oskarsson, Robyn Davies, Lynn Cockburn, Maureen Barry, Aleksandra Bjelajac Mejia, Jose Lanca, Thuan Dao, Judy Watt-Watson, Bonnie Stevens

**Affiliations:** a Department of Pharmacy, University Health Network, Toronto, Ontario, Canada; b Biomedical Communications, Institute of Medical Science, Faculty of Medicine, University of Toronto, Toronto, Ontario, Canada; c University of Toronto Centre for the Study of Pain, University of Toronto, Toronto, Ontario, Canada; d Department of Occupational Science and Occupational Therapy, Faculty of Medicine, University of Toronto, Toronto, Ontario, Canada; e Physician Assistant Program, University of Toronto, Toronto, Ontario, Canada; f Department of Physical Therapy, Faculty of Medicine, University of Toronto, Toronto, Ontario, Canada; g Department of Medicine, Division of Physical Medicine and Rehabilitation, University of Toronto, Toronto, Ontario, Canada; h Toronto Rehabilitation Institute, University Health Network, Toronto, Ontario, Canada; i University of California San Francisco, Zuckerberg San Francisco General, San Francisco, California, USA; j Bridgepoint Health, Sinai Health System, Toronto, Ontario, Canada; k Lawrence S. Bloomberg Faculty of Nursing, University of Toronto, Toronto, Ontario, Canada; l Leslie Dan Faculty of Pharmacy, University of Toronto, Toronto, Ontario, Canada; m Faculty of Dentistry, University of Toronto, Toronto, Ontario, Canada; n Faculty of Medicine, University of Toronto, Toronto, Ontario, Canada

**Keywords:** pain, opioids, interprofessional, curriculum, mapping, health professions, education

## Abstract

**Background:**

There is a growing societal need for health professional competency in pain care. The University of Toronto Centre for the Study of Pain–Interfaculty Pain Curriculum (UTCSP-IPC) has been offered since 2002. Content and process have been updated annually. In addition, participating health professions programs have advanced their pain teaching. A curricular scan was needed to creatively and constructively advance the UTCSP-IPC.

**Aim:**

The aim of this study was to map curricular pain content in participating health professions programs onto the UTCSP-IPC content as a first step to further curriculum design.

**Methods:**

UTCSP-IPC committee members and faculty representatives from six health profession programs completed a 27-item online survey in this collaborative action study. Descriptive statistics were completed in Microsoft Excel.

**Results:**

The UTCSP-IPC provided an average of 43.3% (range 32%–62%) of total pain content teaching hours to participating health professions students and a range of 8% to 100% of total opioid-related teaching hours. Curricular overlaps and gaps in pain content were identified and will be used to update and inform the iterative design of the UTCSP-IPC. Ninety-three percent of participating health professions faculty indicated that the interprofessional focus on pain care in the UTCSP-IPC was important.

**Conclusion:**

This study highlighted the value of the UTCSP and areas of curricular refinement to ensure continued relevance in relationship to pain content within the six participating health professions programs. Mapping a coordinated approach between uniprofessional and interprofessional teaching will both meet the demands of professional competence and create greater applicability to future practice settings.

## RÉSUMÉ

**Contexte** : Il est de plus en plus nécessaire que les professionnels de la santé aient les compétences nécessaires pour la prise en charge de la douleur au sein de la société. Le Programme interfacultaire sur la douleur du Centre pour l’étude de la douleur de l’Université de Toronto (UTCSP-IPC) est offert depuis 2002. Son contenu et son processus ont été mis à jour chaque année. Les programmes des professions du domaine de la santé participantes ont eux aussi perfectionné leur enseignement sur la douleur. Une radiographie du programme était donc nécessaire afin d’améliorer l’UTCSP-IPC de manière créative et constructive.

**Objectif** : Recenser le contenu sur la douleur dans les programmes des professions du domaine de la santé participantes afin de l’inclure dans l’UTCSP-IPC en tant que première étape pour améliorer la conception du programme.

**Méthodes** : Des membres du comité de l’UTCSP-IPC et des représentants des facultés de six programmes de professions du domaine de la santé ont répondu à un sondage en ligne comprenant 27 énoncés dans le cadre de cette étude collaborative. Des statistiques descriptives ont ensuite été compilées dans Microsoft Excel™.

**Résultats** : Au total, l’UTCSP-IPC a donné en moyenne 43,4 % (entre 32 et 62 %) d’heures d’enseignement sur la douleur aux étudiants des professions du domaine de la santé participantes, et de huit à 100 % d’heures d’enseignement liées aux opioïdes. Les lacunes et les chevauchements dans le contenu portant sur la douleur ont été répertoriés. Cette information sera utilisée pour la mise à jour et la conception itérative de l’UTCSP-IPC. Quatre-vingt-treize pour cent des facultés des professions du domaine de la santé participantes ont souligné l’importance de continuer à mettre l’accent sur l’aspect interprofessionnel de la prise en charge de la douleur dans l’UTCSP-IPC.

**Conclusion** : Cette étude a mis en évidence l’importance de l’UTCSP ainsi que les aspects du programme qui doivent être peaufinés pour maintenir sa pertinence en ce qui concerne le contenu sur la douleur au sein des six programmes des professions du domaine de la santé participantes. L’élaboration d’une approche coordonnée entre l’enseignement uniprofessionnel et l’enseignement interprofessionnel permettra à la fois de répondre aux exigences en matière de compétences professionelles et d’améliorer l’applicabilité dans les futurs milieux de pratique.

## Introduction

Pain is the most common reason that individuals seek health care.^1,2^ There is a growing societal need for health professional competency in pain care and the continued need to improve pain education for health professionals,^3–8^ including the multidimensional nature of pain.^8^ The complexity of pain care often requires a collaborative, interprofessional approach.^2,9^ Rational, multimodal pain management remains an important focus in the current climate of concerns about increased prescription opioid usage and associated harms.^10–12^

Well-designed pain curricula can improve pain knowledge.^13^ Education guidelines for prelicensure pain education have advanced from the original International Association for the Study of Pain (IASP) core curriculum guide^14^ to reflect the need for content on the multidimensional nature of pain.^2,8,15,16^ In 2013, a competency-based pain management guide for prelicensure health care professionals was developed at an interprofessional consensus summit to help bridge the gap between the needs of individuals in pain and interprofessional health care professional teams.^2^ Currently, there are few prelicensure interprofessional learning experiences, despite IASP recommendations within the pain assessment and measurement domain for this collaboration.

The University of Toronto Centre for the Study of Pain–Interfaculty Pain Curriculum (UTCSP-IPC), developed initially in 2002, was based on the IASP core and discipline specific curricula.^13^ It has been offered annually to approximately 1000 students from seven health professions programs (Table 1). The 20-h curriculum is focused on the multidimensional nature of acute and chronic pain and collaborative patient-centered pain care, providing sessions that include facilitated interprofessional small group, case-based discussions; multiprofessional and uniprofessional didactic and panel presentations; and two online modules.^13,17^ The curriculum has been evaluated and updated annually based on results from student pre/post knowledge questionnaires, content and process surveys, and recommendations from clinician-facilitators and UTCSP-IPC committee members. Annual qualitative feedback from faculty and students indicate that pain content within each of the participating health professions programs has also advanced over recent years. After 15 years, the UTCSP-IPC committee deemed that a scan of curricular environments was necessary to compare pain content taught in the UTCSP-IPC and participating health professions programs for refinement of curriculum to continue to provide students with a relevant, value-added interprofessional pain education.

**Table 1.** Demographics of survey participants by program.

	Dentistry	Nursing	OS&OT	Pharmacy	PA	PT
Program length (years)	4	2	2	4	2	2
Year students participate in the UTCSP-IPC	3	2	2	2	2	2
How the survey was completed	Individual	Individual	Group	Individual	Individual	Individual
Number of survey participants (including primary participant)	2	5	4	4	2	2
Number of survey entries	2	5	1	4	2	2

OS&OT = Occupational Science and Occupational Therapy; PA = Physician Assistant; PT = Physical Therapy; UTCSP-IPC = University of Toronto Centre for the Study of Pain–Interfaculty Pain Curriculum.

This study’s aims were to (1) examine and map current pain content in each of the participating health profession programs; (2) compare the uniprofessional content with the UTCSP-IPC content, within IASP pain curriculum domains; and, finally, (3) formally evaluate faculty’s confidential perceptions of UTCSP-IPC curricular content, process, and value of the UTCSP-IPC. Of broader interest is an understanding of how uniprofessional teaching on pain fits with an interprofessional pain curriculum to meet pain-related competency development.

## Materials and methods

### Design

This study was designed as collaborative action research.^18,19^ An online survey was constructed.^20,21^ UTCSP-IPC committee members and health professions programs faculty were surveyed to explore the curricular environments of the UTCSP-IPC and the seven health professions programs that participate annually.

### Participants

Individuals were invited to participate by email if they met inclusion criteria: University of Toronto health professions faculty members who teach or have knowledge of pain and/or opioid-related curricular content or who are members of the UTCSP-IPC committee were invited. “Primary participants” responsible for program curricula were invited from each of the health professions programs. These individuals were also identified as having knowledge of and experience with the UTCSP-IPC as past facilitators for small group sessions or UTCSP-IPC committee members, though this was not an inclusion criterion.

### Materials

A survey of 27 quantitative questions was developed by co-investigators based on IASP Interprofessional Curriculum learning objectives and pain content domains: multidimensional nature of pain, pain assessment and measurement, management of pain, and clinical conditions.^15^ The framework to categorize pain content used by the Johns Hopkins curricular development team was also referenced.^8,16^ The Pain Curricular Mapping Survey was composed of four sections: (1) health professions curricula general information (program specific, required completion by primary participants only); (2) participant demographics; (3) course/module format and content; and (4) attitudes, opinions, and perceptions on pain and opioid curricular gaps and overlaps and the value of the UTCSP-IPC. Within the course/module format and content section, participants were asked to describe the context of pain content on the interprofessional continuum of increasingly complex knowledge and understanding of other professions (i.e., uniprofessional, multiprofessional, interprofessional). The attitudes, opinions, and perceptions section included one Likert-scale question,^21^ one question with a dichotomous scale (yes/no or undecided), and a series of qualitative feedback questions that is summarized elsewhere (unpublished observation). Pain Curricular Mapping Survey questions were reviewed by co-investigators and piloted with the UTCSP-IPC evaluation subcommittee prior to administration. Minor changes in wording were made to questions based on feedback obtained. The survey was programmed in REDCap software, password protected, and administered online via an Internet link.

### Procedures

This study was approved by the University of Toronto Health Sciences Research Ethics Board. Invitations were sent to key individuals responsible for curriculum in each faculty/department by email describing the study and seeking informed consent. Once signed informed consent forms were received, these individuals were designated as primary participants and, in collaboration with the investigators, determined how best to complete the survey on behalf of the UTCSP-IPC and their respective faculties/departments. Primary participants were encouraged to recruit secondary participants (i.e., course coordinators, instructors, lecturers, other faculty members) as needed to complete surveys and optimize data collection. Secondary participants also provided informed consent. The online survey link was provided to participants by email after signed informed consent forms were received by the research team. When the survey was completed by a group and facilitated by a study investigator, they accessed the survey link of the primary participant and a study investigator recorded responses and submitted this file. All survey responses were stored on a password-protected website that could only be accessed by the research team. Support from a study investigator to record responses was provided upon request to all survey respondents. When present, the investigator did not influence any responses. Professional ethics of the academic faculty involved in providing group feedback were relied upon. Individual and collective responses were agreed upon in terms of accuracy and completeness. Participants agreed to share their confidential opinions verbally and the investigator recorded responses. The consent form estimated the anticipated length of time as 1–2 h to complete the survey. Time required to complete the survey was dependent upon the number of courses that the respondent was providing responses for. Two scheduled reminder emails were sent to encourage survey response and completion. Data collection and analysis were completed between November 2015 and July 2016.

### Analysis

Data were automatically collected online in REDCap and were downloaded to Microsoft Excel. Descriptive analyses were performed on quantitative data. Data were analyzed by health professions program and the UTCSP-IPC. Primary participants reviewed results from their respective programs and confirmed accuracy and completeness. Results were compared across health professions programs and to the UTCSP-IPC.

## Results

### Demographics

Nineteen health professions faculty members at the University of Toronto (Dentistry, Nursing, Occupational Science and Occupational Therapy [OS&OT], Pharmacy, Physician Assistant [PA], Physical Therapy [PT]) completed 16 survey responses; the survey was completed as a group of faculty members responsible for teaching pain content or individually by a primary participant seeking input from colleagues. At least one respondent from each health professions program had experience as a UTCSP-IPC committee member and curriculum facilitator. All but one primary participant (Pharmacy) had experience as a UTCSP-IPC committee member and facilitator. Responses reflected curricula of the 2014–2015 academic year. The Faculty of Medicine did not participate because they were engaged in curricular revisions during the study period. Participants representing the UTCSP-IPC committee completed the survey as a group. See Table 1 for more details.

### Pain-related content

Pain-related content was mapped within health professions program curricula, categorized according to IASP Interprofessional Curriculum domains, and compared to content within the UTCSP-IPC. Courses/modules with pain-related content were required by each of the six programs (Table 2). Programs varied as to when required courses with pain-related content are offered by year of program. Some programs (i.e., Nursing and Pharmacy) also offered elective courses/modules with pain-related content to senior students. The number of pain content hours mapped by year (Table 3) also was variable among programs. For example, Nursing and PA students received the majority (88% and 89%, respectively) of their pain-related content in year 1 of their 2-year program, whereas pain content hours for Dentistry and OS&OT are weighted heavier later in their programs (i.e., 69% of content in Dentistry is offered in year 3 or 4, 100% in OS&OT is offered in year 2). The PT program had equal distribution of pain-related content between both years of their 2-year program. The Pharmacy program pain-related content hours were distributed over the first 3 years of their curriculum, with 50% offered in year 3. Teaching hours for Pharmacy and Nursing in Table 3 underestimate hours devoted to pain content because selective and elective courses are excluded.

**Table 2.** Number of required and elective courses/modules with pain-related content by program.

	Dentistry	Nursing	OS&OT	Pharmacy	PA	PT
Number of REQUIRED courses/modules with pain-related content						
In year 1	1	6	0	3	9	2
In year 2	1	0	3	5	2	2
In year 3^a^	3			3		
In year 4^a^	2			N/A^b^		
Number of ELECTIVE courses/modules with pain-related content						
In year 2	0	2	0	0	0	0
In year 3	0			8		

^a^Nursing, OS&OT, PA, and PT are 2-year programs.

^b^Year 4 of Pharmacy is exclusively clinical rotations, which were excluded.

OS&OT = Occupational Science and Occupational Therapy; PA = Physician Assistant; PT = Physical Therapy; N/A = not applicable.

**Table 3.** Number of required course/module hours with pain-related content by program and year.

	Dentistry	Nursing	OS&OT	Pharmacy	PA	PT
Number of required course/module hours with pain-related content						
Year 1	7.5	15	0	6	25	20
Year 2	9	2	12	5	3	21
Year 3^a^	9.75			11		
Year 4^a^	27.75			N/A^b^		

^a^Nursing, OS&OT, PA, and PT are 2-year programs.

^b^Year 4 of Pharmacy is exclusively clinical rotations, which were excluded.

OS&OT = Occupational Science and Occupational Therapy; PA = Physician Assistant; PT = Physical Therapy; N/A = not applicable.

All UTCSP-IPC learning objectives were mapped onto those of the IASP Interprofessional Curriculum and the degree to which these were reflected in the objectives of individual programs was examined (Table 4). To compare pain-related content in more detail, subtopics of each of the four IASP Interprofessional Curriculum domains were mapped within each health professions program and the UTCSP-IPC (Table 5). Details of the mapping of other domains are available as a supplementary table. Similar pain curricular domain subtopics were identified between the UTCSP-IPC and across programs, although consistency across programs was lacking as some were covered in only one program (Table 6). Opioid-related content was provided by Dentistry, Nursing, Pharmacy, and PA programs.

**Table 4.** Mapping learning objectives of health professions programs onto those of the UTCSP-IPC and the IASP Interprofessional Curriculum.

IASP Interprofessional Curriculum learning objectives	UTCSP-IPC Program learning objectives	Dentistry	Nursing	OS&OT	Pharmacy	PA	PT
Discuss the multidimensional nature of pain and its components, implications for patients/families, and relationship to clinical interventions.	Relate the basic science of acute and persistent pain to clinical interventions.	Y	Y	Y	Y	Y	Y
	Describe the prevalence and impact of unrelieved pain on individuals, families, and societies.	Y	Y	Y	Y	Y	Y
Discuss inadequately managed pain assessment and management from an ethical, safety, social, and political perspective.	Describe the impact of ethical, legal, social, and political challenges on patients’ pain assessment and management.	Y	N	N	Y	N	Y
Discuss clinical assessment and measurement approaches and misbeliefs common to health care professionals.	Complete a comprehensive assessment of the multiple factors that contribute to the pain experience, including evaluation of pathological sources, possible underlying neurophysiological pain mechanisms, impairment, activity limitation, participation restriction, psychosocial factors.	Y	Y	Y	Y	Y	Y
Develop and discuss as part of an interprofessional student group the rationale for patient-focused pain assessment and management plans based on authentic patient cases (actual or scenarios).	Describe the role of the person in pain in the interprofessional team.	Y	Y	Y	Y	N	Y
	Present a comprehensive pain management plan and justify the choice of interventions, including physical, physiological, and pharmaceutical.	Y	Y	Y	Y	Y	Y
Describe multiprofessional and interprofessional strategies for the planning, intervention, and monitoring of pain management outcomes.	Describe multiprofessional and interprofessional strategies for the planning, intervention, and monitoring of pain management outcomes.	Y	N	N	Y	N	Y

UTCSP-IPC = University of Toronto Centre for the Study of Pain–Interfaculty Pain Curriculum; IASP = International Association for the Study of Pain; OS&OT = Occupational Science and Occupational Therapy; PA = Physician Assistant; PT = Physical Therapy.

**Table 5.** Multidimensional nature of pain covered in each program compared to the UTCSP-IPC.

Multidimensional nature of pain	UTCSP-IPC	Dentistry	Nursing	OS&OT	Pharmacy	PA	PT
Epidemiology of acute and/or persistent pain	Y	Y	Y	Y	Y	N	Y
Consequences of pain as a public health problem (e.g., economic, social, ethical, legal impact)	Y	Y	Y	N	Y	N	Y
Theories and science for understanding pain	Y	Y	Y	N	N	Y	Y
Terminology for describing pain and associated conditions	Y	Y	Y	Y	Y	Y	Y
Influences that affect assessment and management of pain (e.g., patient, provider, cultural, institutional, societal, and regulatory)	Y	Y	Y	Y	Y	Y	Y
Pain mechanisms; anatomy and physiology to include neural mechanisms	Y	Y	Y	Y	N	Y	Y
Multiple dimensions of pain (e.g., physiological, sensory, affective, cognitive, affective, behavioral)	Y	Y	Y	Y	Y	Y	Y
Neurophysiological consequences of unrelieved pain	Y	Y	Y	N	Y	N	Y
Factors influencing neurophysiology (e.g., genetics, sex, age)	Y	Y	Y	N	N	N	Y

UTCSP-IPC = University of Toronto Centre for the Study of Pain–Interfaculty Pain Curriculum; IASP = International Association for the Study of Pain; OS&OT = Occupational Science and Occupational Therapy; PA = Physician Assistant; PT = Physical Therapy.

**Table 6.** Curricular content overlaps and gaps between the UTCSP-IPC and all health professions programs.

IASP Interprofessional Curriculum domain	Curricular overlap between UTCSP-IPC and all health professions programs	Curricular gaps within UTCSP-IPC
Multidimensional nature of pain	Terminology for describing pain and associated conditions Influences that affect assessment and management of pain Multiple dimensions of pain (e.g., physiological, sensory, affective, cognitive, affective, behavioral)	
Pain assessment and measurement	Comprehensive pain assessment (e.g., history, patient expectations, clinical record review) Quantitative and qualitative measures that are reliable and valid	Physical examination (e.g., neurological and musculoskeletal assessment, posture, range of motion) Investigations (e.g., laboratory, imaging)
Management of pain	Goals of management approaches involving patient Type and multidimensional nature of pain; issues related to patient, caregiver, health professional, political context/substance abuse issues Selected nonpharmacological strategies (e.g., clinician therapeutic use of self, health promotion, and self-management)	Selected nonpharmacological strategies (e.g., neuroablative strategies, procedural/interventional, surgery) Selected pharmacological strategies (e.g., local anesthetics, topical agents, cannabinoids, medical marijuana)
Clinical conditions	Pain in selected special populations (e.g., pediatrics) Selected pain conditions (e.g., headache, rheumatoid arthritis, osteoarthritis)	Pain in selected special populations (e.g., older adults, pregnancy, inability to communicate, mental health, substance use disorders, palliative) Selected pain conditions (infection, burn, end-of-life, cardiac and non-cardiac chest pain, abdominal, peritoneal, retroperitoneal pain, pelvic pain, sickle cell crisis, multiple sclerosis, post-stroke, spinal cord injury, degenerative disc disease/acute disc herniation with radiculopathy, peripheral neuropathies, postherpetic neuralgia, complex regional pain syndrome, irritable bowel syndrome, fibromyalgia, low-back pain)

UTCSP-IPC = University of Toronto Centre for the Study of Pain–Interfaculty Pain Curriculum; IASP = International Association for the Study of Pain.

### Time devoted to pain-related content

Including the 20 h of pain-related content provided by the UTCSP-IPC, pain-related content hours within the six health professions programs ranged from 32.25 h for OS&OT to 61.85 h for Pharmacy (Table 7). These hours included required and elective courses/modules. The UTCSP-IPC provided an average of 43.3% (range 32%–62%) of total pain-related content hours to health professions students. The UTCSP-IPC provided a range of 8% to 100% of opioid-related teaching to health professions students in the interprofessional context (Table 8). Opioid-related content represented 5% of total pain-related content within the UTCSP-IPC.

**Table 7.** Teaching hours devoted to pain content.

	Dentistry	Nursing	OS&OT	Pharmacy	PA	PT
Health professions program pain content (hours)	36.75	16	12.25	41.85	28.75	40.5
UTCSP-IPC pain content (hours)	20	20	20	20	20	20
Total pain-related content (hours)	56.75	36	32.25	61.85	48.75	60.5
% content hours provided by UTCSP-IPC	35%	56%	62%	32%	41%	33%

OS&OT = Occupational Science and Occupational Therapy; PA = Physician Assistant; PT = Physical Therapy; UTCSP-IPC = University of Toronto Centre for the Study of Pain–Interfaculty Pain Curriculum.

**Table 8.** Teaching hours devoted to opioid content.

	Dentistry	Nursing	OS&OT	Pharmacy	PA	PT
Health professions program opioid content (hours)	5.5	0.34	0	10.88	3	0
UTCSP-IPC opioid content (hours)	1	1	1	1	1	1
Total opioid-related content (hours)	6.5	1.34	1	11.88	4	1
% content hours provided by UTCSP-IPC	15	75	100	8.4	25	100

OS&OT = Occupational Science and Occupational Therapy; PA = Physician Assistant; PT = Physical Therapy; UTCSP-IPC = University of Toronto Centre for the Study of Pain–Interfaculty Pain Curriculum.

### Teaching and learning

In the UTCSP-IPC, patient-centered pain care was the focus of the curriculum. Only Dentistry, Nursing, and Pharmacy reported having courses/modules in their curricula where pain was the primary focus. All other pain-related content was embedded within other courses and modules (e.g., focused on special populations such as older adults, pediatric, mental health, substance use disorders, childbearing, women/families), clinical conditions (e.g., neurological injuries, oncology, musculoskeletal). The context of pain-related content within the interprofessional continuum across programs was examined.^22^ Pain-related content was taught to students from all faculties and programs interprofessionally in the UTCSP-IPC. PT also provided pain-related content in an interprofessional context outside of the UTCSP-IPC. Nursing and OS&OT provided some pain-related content multiprofessionally, whereas in the Pharmacy and the PA programs, all pain-related content outside of the UTCSP-IPC was taught uniprofessionally.

### Faculty perceptions

Health professions faculty were asked to rate the importance for health professions students to learn about pain in an interprofessional context on a five-point Likert scale from *not very important* (1) to *very important* (5). Almost all respondents (93%) believed this to be important or very important. Most health professions faculty (77%) perceived the UTCSP-IPC as a value-added learning experience for students. Two health professions faculty were undecided and one responded negatively; related feedback expressed concerns about repetition of selected pain content (unpublished observation).

## Discussion

Pain content was categorized within the IASP Interprofessional Curriculum domains and compared across six health professions curricula and with the UTCSP-IPC in this collaborative action study.

Curricular areas of overlap in pain-related content were identified in each of the four IASP interprofessional domains among UTCSP-IPC and uniprofessional health professions programs. Some of these topics were quite broad—for example, terminology for describing pain, valid and reliable tools for measuring pain, goals of management approaches, quantitative and qualitative measures, pain in selected special populations (pediatrics)—but the depth of content provided in these areas remains unclear. Overlap of these topics was value-added, considering their breadth and importance as foundational content. Curricular gaps in domains of the IASP Interprofessional Curriculum were identified within the UTCSP-IPC; for example, physical examination, selected pharmacological and nonpharmacological strategies, and selected special populations (i.e., older adults, pregnancy). Variability exists; some uniprofessional programs provided selected content. For example, physical examination and pain in older adults were covered by six health professions programs; pain in pregnancy was only covered by Nursing, PA, and PT; and medical cannabis was only covered by Pharmacy. In contrast, the UTCSP-IPC was the only source of selected pain content for students from some programs; for example, epidemiology of acute and/or persistent pain and consequences of pain as a public health problem were not covered in the PA program outside of the UTCSP-IPC. Theories and science for understanding pain and factors influencing neurophysiology were not covered in the Pharmacy program. Similarly, the OS&OT program did not cover consequences of pain as a public health problem, theories and science for understanding pain, or factors influencing neurophysiology. Results of this study should prompt participating health professions programs to consider opportunities for review of their own pain curriculum, to ensure a good fit to prepare students for and complement the UTCSP-IPC.

Teaching hours devoted to pain content, when compared with the 2009 survey by Watt-Watson et al., indicate that there was a trend toward increased pain-related teaching hours in Dentistry and Pharmacy programs.^4^ Total pain content teaching hours reported by Dentistry (36.75 h) and Pharmacy (41.85 h) in our study exceeded the highest end of the ranges from the survey in 2009 for these respective professions (24 and 33 h, respectively). It is possible that this is due to differences in designation of formal teaching hours by participants. Despite reports that pain content increased in uniprofessional health professions programs over time, UTCSP-IPC provided an average of 43.3% (range 32%–62%) of total pain content hours of six health professions programs. A need for increased competency in opioid management by health care professionals has been identified in the literature to help cope with what has been described as the current “opioid crisis.”^23^ Although not all professions are prescribers, each has a role to play related to opioids. The UTCSP-IPC provided a range of 8% to 100% of opioid-related teaching hours as a novel 1-h interactive online module for the health professions programs surveyed. Results found that time spent on opioid-related content across health professions programs varied widely, likely due to the differing roles related to opioids in practice (e.g., prescribing, dispensing, administering, or monitoring). The UTCSP-IPC promotes a patient-centered focus and development of interprofessional care plans, balancing physical, psychological, and pharmacological strategies. Therefore, opioid-related content remained a small component (5%) of total pain-related teaching hours within the UTCSP-IPC. A critical finding was that pain was not taught interprofessionally within most of the participating health professions programs. The complexity of pain care demands that health care professionals are competent in a collaborative patient-centered, multidimensional approach. One of the learning objectives of the IASP Interprofessional Curriculum, “Describe multiprofessional and interprofessional strategies for the planning, intervention and monitoring of pain management outcomes,” addresses this need. Results from our survey indicated that outside of the UTCSP-IPC, Nursing, OS&OT, and PA students would not meet this learning objective. The interprofessional approach of the UTCSP-IPC provided all participating programs with this value-added educational experience. Ultimately, when health professions faculty respondents were asked to rate the importance for students to learn about pain in an interprofessional context, 93% indicated that they believed that this was important/very important.

The findings of this study reinforced the value of the UTCSP-IPC and will be used for iterative design and formative feedback purposes to enhance pain content within future iterations. These findings also allowed for a better understanding of how uniprofessional teaching on pain management fits within an interprofessional pain curriculum to meet pain-related competencies. Topics identified as not being covered in the UTCSP-IPC and a majority of health professions programs—for example, pain in mental health and medical cannabis—are planned as future topics for multiprofessional lectures in the next UTCSP-IPC. Given the logistic challenges of providing an effective and engaging curriculum for approximately 1000 interprofessional students and the changing needs of these students over time, exploration of novel methods for teaching and learning is required. Two online modules created in dynamic platforms, “Pain: Mechanisms and Manifestations” and “Opioids As a Component of Pain Management, an Interprofessional Responsibility,” have been implemented in the last few years of the UTCSP-IPC. Further changes and updates to other aspects of the curriculum are planned, including an interactive online format to facilitate case discussion in small groups.

The authors acknowledge limitations of this study. The Faculty of Medicine was unable to participate in this survey and, as a large contributor of resources to the UTCSP-IPC as well as faculty and student participants, inclusion of their results is missed. Although primary responders were selected in this study for their broad perspective on pain teaching in each health professions program, it is possible that the reporting was summarized and not reflective of all details. There was a potential for bias in the selection of the primary participants. They have chosen leadership roles and have self-selected to be involved in the UTCSP-IPC because they are interested in and supportive of interprofessional pain education. Potential bias in faculty involved in the UTCSP-IPC during participation may be a limitation; however, it was mitigated by evidence that could be confirmed through course documents, if needed. Social desirability of responses may be a limitation in a study of this nature. Examination of pain content and learning during clinical rotations was beyond the scope of the study. There is variability across clinical placements and uniprofessional requirements, and between clinical educators. Performing content validity could have strengthened the survey instrument. Some survey questions may have lacked clarity in terminology—for example, *exposure* vs. *skill*—and could be interpreted differently across professions. Particular survey questions presented inherent challenges to the UTCSP-IPC committee; for example, concurrent selective sessions were not included as part of the survey response but provide valuable content in the UTCSP-IPC. One of the strengths of UTCSP-IPC is case-based small group learning with several different cases. Students cover content pertaining to their assigned case topics in greater depth, potentially leading to variations in learning.

## Conclusion

In conclusion, the pain curricular mapping study compared the pain content of six health professions program curricula and the UTCSP-IPC to the IASP Interprofessional Curriculum. Based on the findings from this study, the UTCSP-IPC committee will focus refinement of the curriculum on addressing gaps and incorporating relevant issues for improvement to continue to provide students with a value-added interprofessional pain education experience. Future steps will involve advancement of assessment pertaining to pain competencies in uniprofessional program content. These results will be of interest to health profession programs teaching pain care and those interested in developing pain education in an interprofessional context.

With the need for enhanced pain education recognized among health profession programs, faculty members will need to consider both content and approaches to best meet recommended competencies. A coordinated approach between uniprofessional and interprofessional teaching will both meet the demands of the profession and create greater applicability to future practice settings. The complexity of pain care requires competencies in both profession- and team-based approaches.

## Supplementary Material

Supplemental Material

## Supplementary material

Supplemental data for this article can be access on the publisher’s website.
