# Supplementary material for: Mapping of pain curricula across health professions programs at the University of Toronto
Source: Can J Pain. 2018 Jul 19;2(1):182–90. doi: 10.1080/24740527.2018.1479841 (PMC8730574; doi:10.1080/24740527.2018.1479841)
Supplement: Supplemental Material [file UCJP_A_1479841_SM2866.docx]

Supplementary Tables: IASP Interprofessional Pain Curriculum Domain Subtopics within each Health Professions Program Compared to the UTCSP-IPC

|  | UTCSP-IPC | Dent | Nurs | OS& OT | Pharm | PA | PT |
| --- | --- | --- | --- | --- | --- | --- | --- |
| **Domain: Pain assessment and measurement** |  |  |  |  |  |  |  |
| Interprofessional and multiprofessional collaboration | Y | Y | Y | Y | Y | N | Y |
| Comprehensive pain assessment (e.g. history, patient expectations, clinical record review) | Y | Y | Y | Y | Y | Y | Y |
| Physical examination (e.g. neurological and musculoskeletal assessment, posture, range-of-motion) | N | Y | Y | Y | Y | Y | Y |
| Investigations (e.g. laboratory, imaging) | N | Y | N | N | Y | Y | Y |
| Quantitative and qualitative measures that are reliable and valid, appropriate for the age and condition of the patient. | Y | Y | Y | Y | Y | Y | Y |
| **Domain: Management of pain** |  |  |  |  |  |  |  |
| Goals of management approaches involving patient/family in planning decisions and encourage combination of methods | Y | Y | Y | Y | Y | Y | Y |
| Type and multidimensional nature of pain, issues related to patient, caregiver, health professional, political context as well as substance abuse issues | Y | Y | Y | Y | Y | Y | Y |
| Evaluation of outcomes, monitoring | Y | N | Y | Y | Y | N | Y |
| Clarification of physical dependence, tolerance, aberrant behaviour, substance use disorder, addiction | Y | Y | Y | N | Y | Y | Y |
| Assessment/screening for risk of abuse (e.g. Opioid risk tool, Current opioid misuse measure) | Y | Y | Y | N | Y | N | Y |
| Non-pharmacological strategies: clinician therapeutic use of self (e.g. active-listening, empathy) | Y | Y | Y | Y | Y | Y | Y |
| Non-pharmacological strategies: health promotion and self-management | Y | Y | Y | Y | Y | Y | Y |
| Non-pharmacological strategies: physical strategies to support home and occupational function (e.g. heat, cold, positioning, exercise, massage, wound support, manipulation) | Y | Y | Y | Y | Y | N | Y |
| Non-pharmacological strategies: psychological and behavioural strategies (e.g. cognitive-behavioural strategies, coping, biofeedback, mindfulness, relaxation) | Y | Y | Y | Y | Y | N | Y |
| Non-pharmacological strategies: neuromodulation (e.g. TENS, acupuncture, brain and spinal cord stimulation) | Y | Y | Y | Y | Y | N | Y |
| Non-pharmacological strategies: neuroablative strategies (e.g. neurolytic nerve blocks, neurosurgical techniques) | N | Y | Y | N | N | N | Y |
| Non-pharmacological strategies: procedural/interventional (e.g. injections) | N | Y | Y | N | Y | Y | Y |
| Non-pharmacological strategies: surgery | N | Y | Y | N | N | Y | Y |
| Pharmacological strategies (analgesics and adjuvants): acetaminophen | Y | Y | Y | N | Y | Y | Y |
| Pharmacological strategies (analgesics and adjuvants): non-steroidal anti-inflammatory drugs (NSAIDS)/COX-2 inhibitors | Y | Y | Y | N | Y | Y | Y |
| Pharmacological strategies (analgesics and adjuvants): opioids | Y | Y | Y | N | Y | Y | Y |
| Pharmacological strategies (analgesics and adjuvants): antidepressants | Y | Y | Y | N | Y | Y | Y |
| Pharmacological strategies (analgesics and adjuvants): anticonvulsants | Y | Y | Y | N | Y | Y | Y |
| Pharmacological strategies (analgesics and adjuvants): local anesthetics | N | Y | Y | N | Y | Y | Y |
| Pharmacological strategies (analgesics and adjuvants): topical agents | N | Y | Y | N | Y | Y | N |
| Pharmacological strategies (analgesics and adjuvants): cannabinoids | N | Y | N | N | Y | N | N |
| Pharmacological strategies (analgesics and adjuvants): medical cannabis | N | N | N | N | Y | N | N |
| Pharmacological strategies (analgesics and adjuvants): natural health products | Y | N | N | N | Y | N | N |
| Pharmacological strategies (analgesics and adjuvants): co-analgesics | Y | Y | Y | N | Y | N | Y |
| **Domain: Clinical conditions** |  |  |  |  |  |  |  |
| Taxonomy of pain systems: type of pain (e.g. acute, recurrent, incident, persistent (chronic) pain) | Y | Y | Y | N | Y | Y | Y |
| Taxonomy of pain systems: nociceptive and neuropathic pain | Y | Y | Y | N | Y | Y | Y |
| Taxonomy of pain systems: commonly used pain terms (e.g. allodynia, analgesia, dysesthesia, hyperalgesia, paresthesia) | Y | Y | Y | N | Y | Y | Y |
| Pain in special populations: pediatrics | Y | Y | Y | Y | Y | Y | Y |
| Pain in special populations: older adults | N | Y | Y | Y | Y | Y | Y |
| Pain in special populations: pregnancy | N | N | Y | N | N | Y | Y |
| Pain in special populations: inability to communicate | N | N | Y | Y | Y | N | N |
| Pain in special populations: mental health | N | Y | N | N | N | N | N |
| Pain in special populations: substance use disorders | N | Y | N | N | Y | N | N |
| Pain in special populations: palliative | N | N | N | N | Y | N | Y |
| Pain conditions: surgery | Y | Y | Y | N | Y | Y | Y |
| Pain conditions: trauma | Y | Y | Y | N | Y | Y | Y |
| Pain conditions: infection | N | Y | Y | N | Y | Y | Y |
| Pain conditions: inflammation | Y | Y | Y | N | Y | Y | Y |
| Pain conditions: burn | N | N | N | Y | N | Y | Y |
| Pain conditions: cancer (e.g. primary pain, local invasion, metastatic spread) | Y | Y | Y | N | Y | N | N |
| Pain conditions: end-of-life | N | N | N | Y | Y | N | Y |
| Pain conditions: referred visceral pain | Y | N | Y | Y | Y | Y | N |
| Pain conditions: cardiac and non-cardiac chest pain | N | Y | Y | Y | Y | Y | N |
| Pain conditions: abdominal, peritoneal, retroperitoneal pain | N | N | Y | N | Y | Y | N |
| Pain conditions: pelvic pain | N | N | Y | N | Y | Y | N |
| Pain conditions: sickle cell crisis | N | N | N | N | Y | Y | N |
| Pain conditions: headache | Y | Y | Y | Y | Y | Y | Y |
| Pain conditions: facial (e.g. orofacial, trigeminal neuralgia) | Y | Y | Y | N | Y | Y | Y |
| Pain conditions: multiple sclerosis | N | N | Y | N | N | N | N |
| Pain conditions: post-stroke | N | N | Y | Y | N | N | Y |
| Pain conditions: spinal cord injury | N | N | N | N | N | N | Y |
| Pain conditions: traumatic brain injury | Y | N | Y | Y | Y | N | Y |
| Pain conditions: degenerative disc disease/acute disc herniation with radiculopathy | N | N | N | N | N | Y | Y |
| Pain conditions: peripheral neuropathies | N | N | Y | N | Y | Y | Y |
| Pain conditions: post herpetic neuralgia | N | N | Y | N | N | Y | N |
| Pain conditions: complex regional pain syndrome | N | N | N | Y | N | N | Y |
| Pain conditions: phantom limb | Y | Y | N | N | N | N | Y |
| Pain conditions: irritable bowel syndrome | N | N | N | N | N | Y | N |
| Pain conditions: fibromyalgia | N | N | N | N | N | Y | Y |
| Pain conditions: rheumatoid arthritis | Y | Y | Y | Y | Y | Y | Y |
| Pain conditions: osteoarthritis | Y | Y | Y | Y | Y | Y | Y |
| Pain conditions: neck pain, whiplash | Y | N | Y | N | N | Y | Y |
| Pain conditions: low back pain | N | N | Y | N | Y | Y | Y |
| Pain conditions: myofascial pain | Y | Y | Y | N | N | Y | Y |

*NOTE: Dent=Dentistry, N=No, Nurs=Nursing, OS&OT=Occupational Science and Occupational Therapy, Pharm=Pharmacy, PA=Physician Assistant, PT=Physical Therapy, Y=Yes*
